# The cytotoxicity of γ-secretase inhibitor I to breast cancer cells is mediated by proteasome inhibition, not by γ-secretase inhibition

**DOI:** 10.1186/bcr2347

**Published:** 2009-08-06

**Authors:** Jianxun Han, Ivy Ma, Michael J Hendzel, Joan Allalunis-Turner

**Affiliations:** 1 Department of Oncology, University of Alberta, Cross Cancer Institute, 11560 University Avenue, Edmonton, Alberta, Canada T6G 1Z2

## Abstract

**Introduction:**

Notch is a family of transmembrane protein receptors whose activation requires proteolytic cleavage by γ-secretase. Since aberrant Notch signaling can induce mammary carcinomas in transgenic mice and high expression levels of Notch receptors and ligands correlates with overall poor clinical outcomes, inhibiting γ-secretase with small molecules may be a promising approach for breast cancer treatment. Consistent with this hypothesis, two recent papers reported that γ-secretase inhibitor I (GSI I), Z-LLNle-CHO, is toxic to breast cancer cells both in vitro and in vivo. In this study, we compared the activity and cytotoxicity of Z-LLNle-CHO to that of two highly specific GSIs, DAPT and L-685,458 and three structurally unrelated proteasome inhibitors, MG132, lactacystin, and bortezomib in order to study the mechanism underlying the cytotoxicity of Z-LLNle-CHO in breast cancer cells.

**Methods:**

Three estrogen receptor (ER) positive cell lines, MCF-7, BT474, and T47D, and three ER negative cell lines, SKBR3, MDA-MB-231, and MDA-MB-468, were used in this study. Both SKBR3 and BT474 cells also overexpress HER2/neu. Cytotoxicity was measured by using an MTS cell viability/proliferation assay. Inhibition of γ-secretase activity was measured by both immunoblotting and immunofluorescent microscopy in order to detect active Notch1 intracellular domain. Proteasome inhibition was determined by using a cell-based proteasome activity assay kit, by immunoblotting to detect accumulation of polyubiquitylated protein, and by immunofluorescent microscopy to detect redistribution of cellular ubiquitin.

**Results:**

We found that blocking γ-secretase activity by DAPT and L-685,458 had no effect on the survival and proliferation of a panel of six breast cancer cell lines while Z-LLNle-CHO could cause cell death even at concentrations that inhibited γ-secretase activity less efficiently. Furthermore, we observed that Z-LLNle-CHO could inhibit proteasome activity and the relative cellular sensitivity of these six breast cancer cell lines to Z-LLNle-CHO was the same as observed for three proteasome inhibitors. Finally, we found that the cell killing effect of Z-LLNle-CHO could be reversed by a chemical that restored the proteasome activity.

**Conclusions:**

We conclude that the cytotoxicity of Z-LLNle-CHO in breast cancer cells is mediated by proteasome inhibition, not by γ-secretase inhibition.

## Introduction

Notch is a family of single-pass type I transmembrane protein receptors that, in mammals, includes four homologs, Notch1 to 4 [1]. Ligand-induced Notch receptor activation requires at least two cleavages that release the intracellular domain from the cytomembrane and allow it to translocate into the nucleus where it activates its target genes [1]. The final cleavage is performed by γ-secretase, whose substrates include all four Notch receptors and their ligands as well as β-amyloid precursor protein, E-cadherin, CD44, ErbB-4, and ephrin-B1 [2-8].

Aberrant Notch signaling can induce oncogenesis and may promote the progression of breast cancer. Transgenic mice overexpressing active Notch1, Notch3, or Notch4 homologs all developed mammary carcinoma [9,10]. Furthermore, a recent clinical study reported that the expression level of Notch1, Notch3, and JAG-1, one of the Notch ligands, were inversely correlated with the overall clinical outcomes in breast cancer patients [11]. These observations have prompted great interest in targeting Notch signaling in breast cancer for therapeutic benefit. However, it should be noted that Notch2 signaling has been reported to function as a tumor suppressor in breast cancer cells [12].

Among the several options to block Notch signaling, inhibition of γ-secretase by small molecules offers a promising approach and has been used extensively to study the downstream targets of the Notch signaling pathway [13,14]. However, experimental data supporting the concept that γ-secretase inhibitors (GSIs) could inhibit the growth of, or kill, breast cancer cells have been scarce. Two recent reports have provided the strongest evidence by showing that Z-LLNle-CHO, commonly considered to be a GSI, has such an effect both *in vitro *and *in vivo *[15,16].

Proteasome inhibitors are a class of recent developed anticancer drugs. Z-LLNle-CHO, as a derivative of a widely used proteasome inhibitor MG-132, has been reported to inhibit chymotryptic protease activity, a core function of the proteasome [17]. In this study, we compared the activity and cytotoxic effects of Z-LLNle-CHO with those of two other widely used and highly specific GSIs, DAPT and L-685,458, and with those of three structurally unrelated proteasome inhibitors, MG132, lactacystin, and bortezomib. Our results suggest that the cell killing effect of Z-LLNle-CHO is not mediated by γ-secretase inhibition, but is mediated by proteasome inhibition.

## Materials and methods

### Reagents

Z-Leu-Leu-Nle-CHO (Z-LLNle-CHO, also called GSI I), N-(N-(3,5-Difluorophenacetyl-L-alanyl))-S-phenylglycine *t*-Butyl Ester (commonly called DAPT or GSI IX), (1S-Benzyl-4R-(1-(1S-carbamoyl-2-phenethylcarbamoyl)-1S-3-methylbutylcarbamoyl)-2R-hydroxy-5-phenylpentyl) carbamic acid *tert*-butyl ester (commonly called L-685,458 or GSI X), Z-Leu-Leu-Leu-aldehyde (Z-LLL-CHO, commonly referred to as MG132), lactacystin, and edaravone were purchased from Calbiochem (San Diego, CA, USA) and dissolved in dimethyl sulfoxide (DMSO). Bortezomib was purchased from LC Laboratories (Woburn, MA, USA) and dissolved in DMSO. Tiron was from Sigma (St. Louis, MO, USA) and dissolved in water.

### Cell culture

Three estrogen receptor (ER) positive cell lines, MCF-7, T47D, and BT474, and three ER negative cell lines, SKBR3, MDA-MB-231, and MDA-MB-468, were used in this study. Both SKBR3 and BT474 cells also overexpress HER2/neu. The culture medium was DMEM/F-12 medium (Gibco, Carlsbad, CA, USA) supplemented with 10% FBS (Gibco) and GlutaMAX (Gibco) for all cell lines except SKBR3, which was cultured in McCoy's 5A medium (Gibco) supplemented with 10% FBS and GlutaMAX. In addition, MCF-7 culture medium was supplemented with non-essential amino acids (Gibco), sodium pyruvate (Gibco)and 10 μg/ml of insulin (Sigma). T47D culture medium was also supplemented with insulin (10 μg/ml). All cell lines were maintained at 37°C in a humidified atmosphere of 5% carbon dioxide in air.

### Cell viability and proliferation assay

Cell viability and proliferation was measured using the CellTiter 96^® ^AQ_ueous _One Solution Cell Proliferation Assay (MTS) kit (Promega, Madison, WI, USA). Cells (3000 to 8000 cells/well) were seeded into 96-well plates in triplicate and allowed to attach overnight before being treated with increasing concentrations of the drugs. All wells, including the control, were exposed to the same concentration of DMSO to eliminate any possible effect of the vehicle on cell viability and proliferation. MTS reagent (20 μl) was added to each well 72 hours later and, after one to four hours incubation, the absorbance at 490 nm was measured using a microplate reader (FLUOstar OPTIMA from BMG LABTECH, Offenburg, Germany). Relative cell viability and proliferation of individual samples was calculated by normalizing their absorbance to that of the corresponding control sample. The mean and standard deviation (SD) of three independent experiments were used to plot dose-response curves. The concentrations that kill and/or inhibit cell growth by 50% (EC_50_) were calculated from the equations that best fit the linear range of the dose-response curves.

### Protein sample preparation

Cells at 80% confluence were treated overnight with drugs at the indicated concentrations and control cultures received DMSO. The next day, cells were incubated with trypsin/EDTA (Gibco) solution for 10 minutes before collection by centrifugation. Cell pellets were then washed once with ice-cold PBS, lysed in lysis buffer (100 mM Tris-HCl (pH 6.8), 10% glycerol, 2% SDS, 1 mM EDTA, 0.002% bromophenol blue, 2 mM NaF, 1 mM Na_3_VO_4_, 1 × protease inhibitor cocktail (Roche Applied Science, Indianapolis, IN, USA)), boiled for five minutes, and passed through a 21 gauge needle. The positive control samples were prepared in the same way as the GSI-treated samples and the negative control samples were prepared by adding the lysis buffer directly to the culture plates after washing with PBS without trypsin/EDTA incubation. Protein concentrations were quantified using a BCA protein assay (Pierce, Rockland, IL, USA).

### Western blot analysis

Protein samples (50 μg/lane) were separated in 8% SDS-PAGE gels and transferred to Trans-Blot^® ^pure nitrocellulose membranes (0.2 μm, Bio-Rad, Hercules, CA, USA). The membranes were blocked with 5% skim milk in TTBS (0.1% Tween-20, 100 mM Tris-HCl (pH 7.4), 150 mM NaCl) at room temperature for one hour before being probed overnight at 4°C with primary antibody solution. The primary antibodies used were anti-Notch1 (Val1744; Cell Signaling Technology, Danvers, MA, USA, 1:1000), anti-ubiquitin (clone FK2 from Millipore, Billerica, MA, USA, 1:1000) and anti-actin (Abcam, Cambridge, MA, USA, 1:5000). After washing with TTBS four times for 10 minutes each, the membranes were incubated with horseradish peroxidase-conjugated anti-rabbit or anti-mouse (Jackson ImmunoResearch Laboratories, West Grove, PA, USA, 1:15,000) secondary antibody solution at 4°C for three hours. After another round of four washes with TTBS, the membranes were incubated with SuperSignal West Pico Chemiluminescent Substrate (Pierce), exposed to Fuji (Tokyo, Japan) film, and then developed to visualize the protein signal.

### Construction of flag-tagged Notch1 extracellular truncation (N1EXT) vector

Synthetic DNA oligonucleotides corresponding to the cDNA encoding human Notch1 signal peptide flanked by restriction enzyme recognition sequences were integrated into pCMV-Tag4A vector (Stratagene, La Jolla, CA, USA) using Sac II/BamH I sites. Then the cDNA encoding the amino acid residues 1721 to 2555 (corresponding to the substrate of γ-secretase) was amplified using reverse transcription-coupled PCR of MCF-7 total cellular RNA and integrated into the vector containing the Notch1 signal peptide-encoding sequence using BamH I/EcoR I sites. The sequence of the new construct was verified by sequencing using T3/T7 primers.

### Transfection and treatment

N1EXT plasmid DNA was transfected into MCF-7 and SKBR3 cells plated on glass coverslips using Lipofectamine 2000 reagent (Invitrogen, Carlsbad, CA, USA). Culture medium was replaced six hours after transfection with fresh medium containing 5 μM of DAPT, 2 μM of L-685,458, or Z-LLNle-CHO at the calculated EC_50 _values of individual cell lines. After overnight incubation to allow the expression of exogenous protein, cells were fixed with 4% paraformaldehyde solution for indirect immunofluorescent microscopy.

### Indirect immunofluorescent microscopy

Fixed cells were first permeabilized with 0.5% Triton X-100 in PBS at room temperature for five minutes and then probed with anti-Flag monoclonal antibody (clone M2 from Sigma, 1:500) at room temperature for one hour. After five washes with PBS, cells were incubated with Alexa 488-conjugated goat anti-mouse secondary antibody (Molecular Probes, Carlsbad, CA, USA, 1:250) at room temperature for 45 minutes and further counterstained with 0.5 μg/ml of DAPI after five washes with PBS. Images were taken using LSM 510 laser scanning confocal microscope with a Plan-Neofluar 40X/1.3NA oil-immersion objective lens (Carl Zeiss, Jena, Germany). The optical slice thickness was less than 0.9 μm.

### Determination of ubiquitin distribution

MCF-7 and MDA-MB-231 cells plated on glass coverslips were treated with drugs at the indicated concentrations for four hours before being fixed in 4% paraformaldehyde solution. Fixed cells were immunostained in the same way as above except that anti-ubiquitin monoclonal antibody (clone FK2 from Millipore, 1:1,000) was used as the primary antibody. Images were taken using LSM 710 laser scanning confocal microscope with a Plan-Apochromat 20X/0.8NA objective lens (Carl Zeiss). The optical slice thickness was 1.8 μm.

### Proteasome activity assay

Proteasome activity was measured using the Proteasome-Glo™ Chymotrypsin-Like Cell-Based Assay kit (Promega, Madison, WI, USA). Briefly, MCF-7 (6000 cells/well) and MDA-MB-231 (10^4 ^cells/well) cells were plated into white-walled 96-well plates. After overnight incubation to allow cell attachment, cells were treated with drugs at indicated concentrations for two hours. Equal volumes of Proteasome-Glo™ reagent were then added and the luminescence signal was measured using a microplate reader (FLUOstar OPTIMA).

## Results

### Among the three GSIs, only Z-LLNle-CHO induces cell death

**Figure 1:**
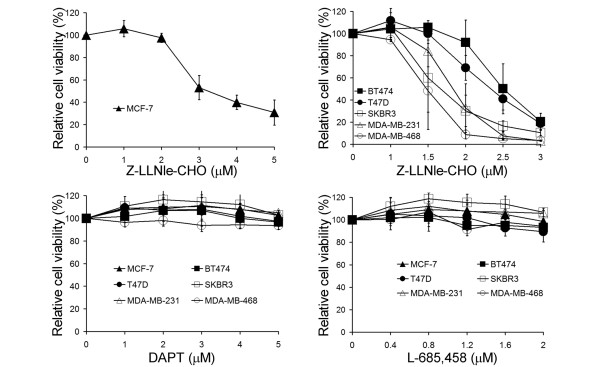
Effect of three different GSIs on the viability/proliferation of six breast cancer cell lines. Breast cancer cells were treated with Z-LLNle-CHO, DAPT, or L-685,458 for 72 hours before relative cell viability/proliferation was measured by MTS assay. Results represent the mean ± standard deviation of three independent experiments. GSI = γ-secretase inhibitor.

We first compared the cytotoxicity of Z-LLNle-CHO to two other widely used GSIs, DAPT and L-685,458. Treatment with Z-LLNle-CHO resulted in a dose-dependent decrease in cell viability/proliferation of all six breast cancer cell lines tested with ER-negative cell lines being more sensitive than ER-positive cell lines. The calculated ED_50 _values were 3.25 μM, 2.5 μM, 2.4 μM, 1.8 μM, 1.6 μM, and 1.4 μM for MCF-7, BT474, T47D, MDA-MB-231, SKBR3, and MDA-MB-468, respectively. However, DAPT and L-685,458 had no cell killing and/or growth inhibitory effects at concentrations up to 5 μM and 2 μM, respectively (Figure 1).

### All three GSIs inhibit γ-secretase activity

**Figure 2:**
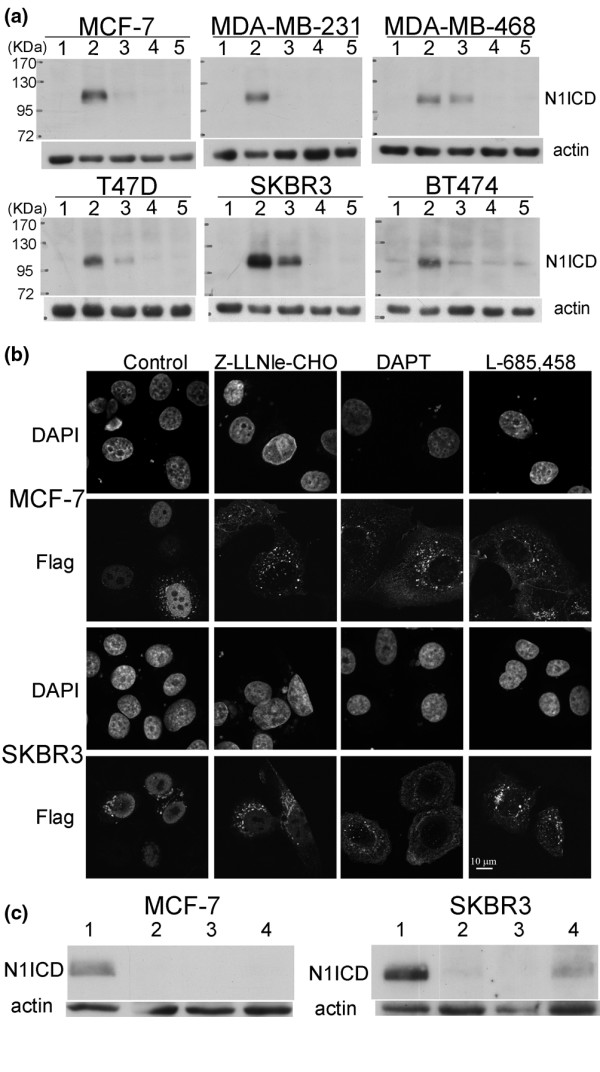
DAPT and L-685,458 inhibit γ-secretase activity. **(a) **Cells were treated overnight with Z-LLNle-CHO at calculated ED_50 _values, 5 μM of DAPT, or 2 μM of L-685,458 before protein samples were prepared. Extracellular calcium was depleted by incubation with 0.53 mM of EDTA for 10 minutes to activate Notch1 before sample preparation for all samples except the negative controls. Protein samples were then subjected to western blot analysis with an antibody (V1744) that specifically recognizes active Notch1 intracellular domain (product of γ-secretase-mediated cleavage). Stronger V1744 signal intensity indicates greater γ-secretase activity. The treatment conditions were (from lane 1 to lane 5): dimethyl sulfoxide (DMSO) vehicle only and without calcium depletion as negative control; DMSO vehicle only and with calcium depletion to activate Notch1 as positive control; Z-LLNle-CHO at concentrations equal to the IC_50 _of individual cell lines; DAPT at 5 μM; L-685,458 at 2 μM. **(b) **MCF-7 (top panels) and SKBR3 (bottom panels) cells were transfected with plasmid DNA expressing a Flag-tagged protein that mimics the immediate substrate of γ-secretase, treated overnight with Z-LLNle-CHO at calculated ED_50 _values, 5 μM of DAPT or 2 μM of L-685,458, and then immunostained with anti-Flag antibody. The appearance of nuclear Flag signal indicates the presence of active γ-secretase. Please note the γ-value of Flag signal was enhanced to visualize weak nuclear or cytomembrane signal. **(c) **Protein samples were prepared without calcium depletion from MCF-7 and SKBR3 cells that were transfected and treated in the same way as the cells in panel b and were subjected to immunoblotting with anti-Notch1 (V1744) antibody. The treatment conditions were (from lane 1 to lane 4): DMSO vehicle only; DAPT; L-685,458; and Z-LLNle-CHO.

We then examined whether the lack of cell killing/growth inhibition by DAPT and L-685,458 was due to their lower potency in inhibiting γ-secretase activity. To address this question, we first performed immunoblot analysis using an antibody that only recognizes cleaved Notch1 intracellular domain (N1ICD) [18,19]. As N1ICD is a product of γ-secretase, its abundance is a good indicator of γ-secretase activity. However, the endogenous N1ICD level (the negative control lanes in Figure 2a) is too low to be detected confidently. Therefore, we took advantage of the fact that calcium depletion activates Notch1 in the absence of ligand binding [20]. As shown in Figure 2a, DAPT at 5 μM and L-685,458 at 2 μM could block calcium depletion-induced Notch1 cleavage in all six cell lines. At the same time, Z-LLNle-CHO, at the concentrations that inhibited cell growth/viability by 50%, failed to do so to a comparable level in SKBR3 and MDA-MB-468 cells, although similar inhibition was observed in the other four cell lines treated with this drug.

To confirm the potency of DAPT and L-685,458 on inhibiting γ-secretase activity in intact cells, we transfected MCF-7 and SKBR3 cells with a plasmid expressing a Flag-tagged N1EXT fragment that mimics the immediate substrate of γ-secretase and then treated them with the same concentrations of GSIs as used for the western blot analysis. Without any intervention, the exogenous protein will be cleaved by γ-secretase as long as it is transported to the plasma membrane to produce N1ICD that can be visualized as nuclear signal when transfected cells are immunostained with an anti-Flag antibody (control panels in Figure 2b). In contrast, when γ-secretase activity is inhibited, the exogenous protein cannot be cleaved and therefore will accumulate at the plasma membrane. As shown in Figure 2b, all the DAPT- and L-685,458-treated cells and Z-LLNle-CHO-treated MCF-7 cells showed exclusively membrane signal. However, 24% and 58% of Z-LLNle-CHO-treated SKBR3 cells displayed mainly nuclear signal or a mixture of nuclear and plasma membrane signal, respectively. This is consistent with the immunoblotting data demonstrating that DAPT and L-685,458 could completely inhibit γ-secretase activity at tested concentrations in both cell lines but Z-LLNle-CHO could only do so in MCF-7 cells (Figure 2c).

Taken together, because complete inhibition of γ-secretase activity by two structurally unrelated GSIs had no effect on cell viability and proliferation, it is unlikely that the cell killing/growth inhibitory effect of Z-LLNle-CHO on breast cancer cell lines was mediated by γ-secretase inhibition.

### Z-LLNle-CHO has proteasome inhibitory activity

**Figure 3:**
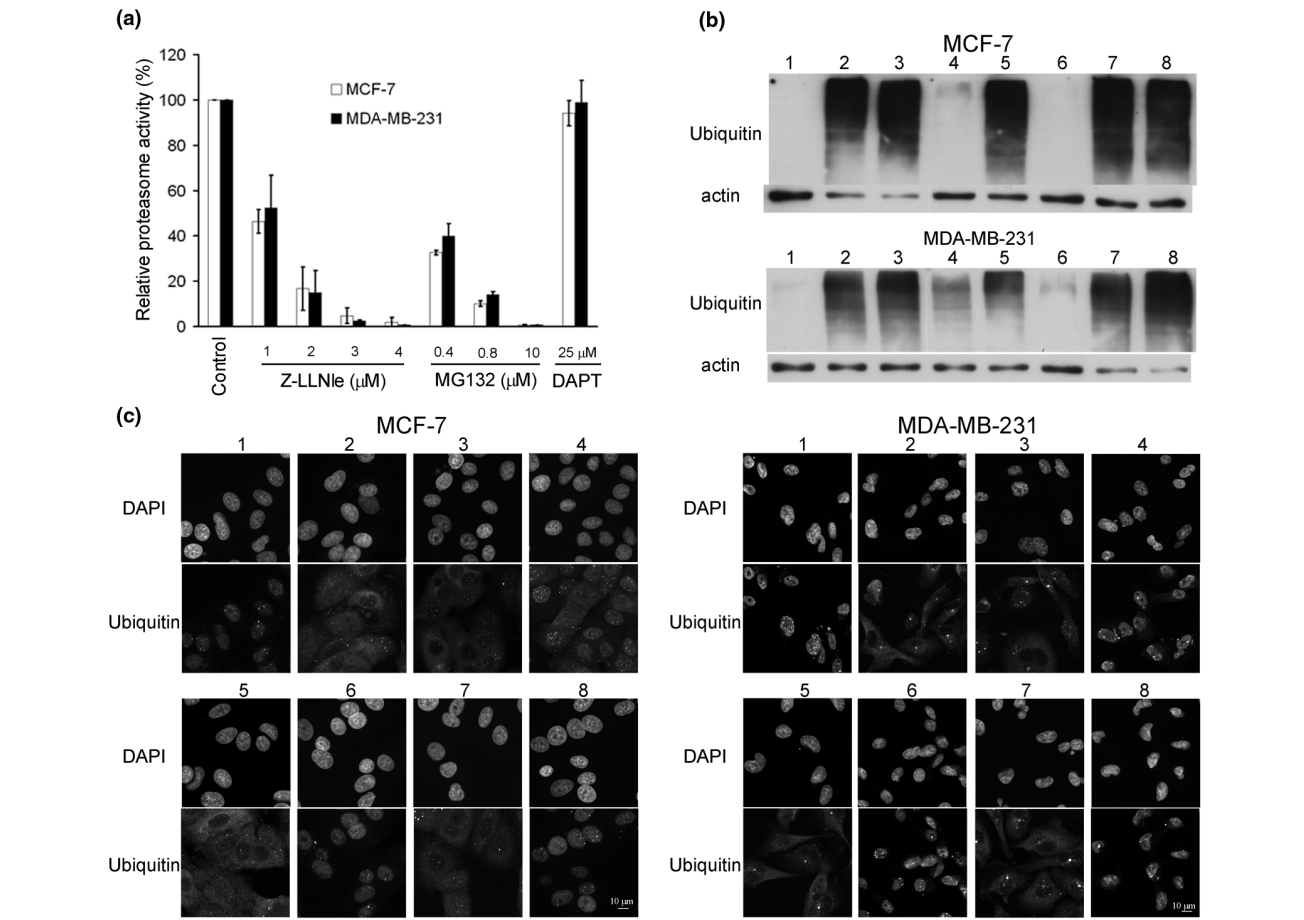
Z-LLNle-CHO has a proteasome inhibitory function. **(a) **Proteasome activity in intact cells was directly measured using a cell-based assay after MCF-7 and MDA-MB-231 cells were treated with indicated drugs for two hours. Results represent the mean ± standard deviation of three independent experiments. **(b) **Protein samples were prepared from MCF-7 and MDA-MB-231 cells that were treated with different combinations of drugs overnight and were subject to immunoblotting with anti-ubiquitin antibody (clone FK2 from Millipore, 1:1,000). Actin immunoblotting was used as loading control. The treatment conditions were (from lane 1 to lane 8): dimethyl sulfoxide (DMSO) vehicle only; Z-LLNle-CHO alone; Z-LLNle-CHO plus tiron; Z-LLNle-CHO plus edaravone; bortezomib alone; bortezomib plus tiron; bortezomib plus edaravone; lactacystin. The concentrations of Z-LLNle-CHO, tiron, edavarone, bortezomib, and lactacystin are 5 μM, 2 mM, 100 μM, 100 nM, 20 μM for MCF-7 cells, and 2.5 μM, 0.5 mM, 100 μM, 40 nM, 5 μM for MDA-MB-231 cells, respectively. The accumulation of polyubiquitinated proteins is an indicator of proteasome inhibition. **(c) **MCF-7 and MDA-MB-231 cells were treated with different combinations of drugs for four hours and then immunostained with anti-ubiquitin FK2 antibody. The treatment conditions were (from 1 to 8): DMSO vehicle only; Z-LLNle-CHO alone; Z-LLNle-CHO plus tiron; Z-LLNle-CHO plus edaravone; bortezomib alone; bortezomib plus tiron; bortezomib plus edaravone; DAPT. The concentrations of Z-LLNle-CHO, tiron, edaravone, and bortezomib were the same as that were used for preparation of protein samples in subsection b. 5 μM of DAPT was used for both MCF-7 and MDA-MB-231 cells. The redistribution of nuclear ubiquitin to cytoplasm is a phenomenon that can be induced by proteasome inhibition.

Z-LLNle-CHO is derived from a widely used proteasome inhibitor MG132 (Z-LLL-CHO) and has been reported to be a broad chymotryptic and aspartyl protease inhibitor [17]. Therefore, we examined whether Z-LLNle-CHO also has proteasome inhibitor activity at the concentrations that showed dose-dependent cytotoxicity. We first used a cell-based proteasome activity kit to measure proteasome activity after cells were treated with MG132, Z-LLNle-CHO, or DAPT. As shown in Figure 3b, both Z-LLNle-CHO and MG132 showed a dose-dependent inhibition of the proteasome at concentrations that showed cytotoxic effects, although DAPT did not. Next, we examined whether or not inhibition of proteasome activity caused accumulation of polyubiquitinated protein, one of the major causes of proteasome inhibitor-induced cell death [21], by subjecting the protein samples from cells treated with 5 μM (MCF-7) or 2.5 μM (MDA-MB-231) of Z-LLNle-CHO overnight to immunoblotting with an anti-ubiquitin antibody. We used bortezomib, a specific proteasome inhibitor that has been approved to treat multiple myeloma in patients, as the positive control. The results showed that treatment with Z-LLNle-CHO indeed resulted in the same accumulation of polyubiquitinated protein that was observed with bortezomib (lane 2 and 5 of Figure 3b). Finally, we took advantage of a recent observation that when proteasome-mediated protein degradation was inhibited, cellular ubiquitin would undergo a nuclear-to-cytoplasmic redistribution that could be detected by anti-ubiquitin FK2 antibody [22]. In untreated MCF-7 and MDA-MB-231 cells, FK2 staining showed dominant nuclear signal (Figure 3c). After a four hour treatment with either bortezomib or Z-LLNle-CHO but not with DAPT, cells displayed a strong cytoplasmic ubiquitin signal, confirming proteasome activity was inhibited by Z-LLNle-CHO.

### The cellular sensitivity of six breast cancer cell lines to Z-LLNle-CHO is the same as that to proteasome inhibitors

**Figure 4:**
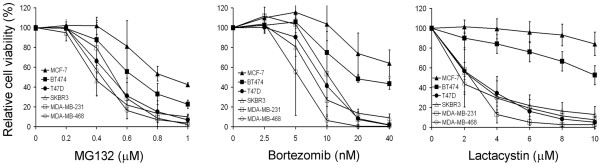
The relative sensitivity of six cell lines to three proteasome inhibitors is the same as that to Z-LLNle-CHO. Cells were treated with MG132, bortezomib, or lactacystin at indicated concentrations for 72 hours before cell viability was measured by MTS assay. Results represent the mean ± standard deviation of three independent experiments. Please note the relative cellular sensitivity of the same six breast cancer cell lines to three structurally unrelated proteasome inhibitors was the same as that to Z-LLNle-CHO in Figure 1.

We next asked whether or not the cell killing effect of Z-LLNle-CHO is mediated by its proteasome inhibition activity. If this is the case, the relative cellular sensitivity of different breast cancer cell lines to Z-LLNle-CHO should reflect that produced by other proteasome inhibitors. Therefore, we treated the same six breast cancer cell lines with increasing doses of three structurally unrelated proteasome inhibitors, MG132, lactacystin, and bortezomib, and measured the effects on cell viability/proliferation using the MTS assay. Similar to the results shown in Figure 1, ER-positive cell lines were more resistant to all the three proteasome inhibitors than ER-negative cell lines (Figure 4). In addition, our results were also consistent with a previous study using bortezomib alone [23]. These data strongly suggest that the cell killing effects of Z-LLNle-CHO in breast cancer cells is mediated by its proteasome inhibitory function.

### The cytotoxicity of Z-LLNle-CHO can be reversed by a specific antioxidant that restores proteasome activity

**Figure 5:**
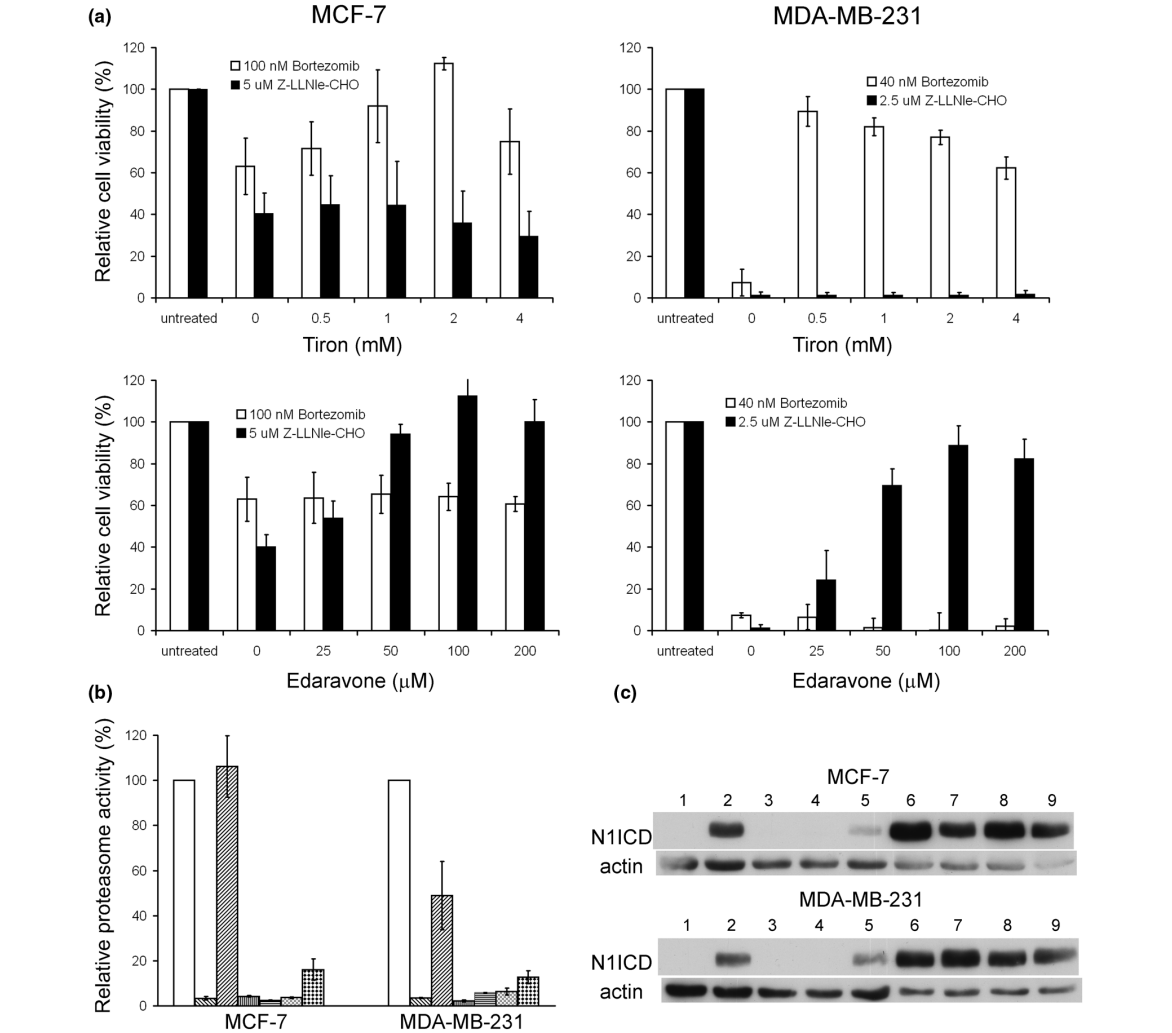
The cytotoxicity effect of Z-LLNle-CHO could be reversed by edaravone that blocks its proteasome inhibitory function. **(a) **Cells were treated with indicated drugs for 72 hours before cell growth was measured using MTS assay. Results represent the mean ± standard deviation (SD) of three independent experiments. **(b) **Proteasome activity in intact cells was directly measured using a cell-based assay after cells were treated with different combinations of drugs for two hours. The treatment conditions were (from left to right): dimethyl sulfoxide (DMSO) vehicle only; bortezomib alone; bortezomib plus tiron; bortezomib plus edaravone; Z-LLNle-CHO alone; Z-LLNle-CHO plus tiron; and Z-LLNle-CHO plus edaravone. The concentrations of bortezomib, tiron, edavarone, and Z-LLNle-CHO are 100 nM, 2 mM, 100 μM, and 5 μM for MCF-7 cells, and 40 nM, 0.5 mM, 100 μM, and 2.5 μM for MDA-MB-231 cells, respectively. Results represent the mean ± SD of three independent experiments. **(c) **The same protein samples used for immunoblotting in Figure 3b plus another negative control sample were subjected to immunoblotting with anti-Notch1 (V1744) antibody that specifically recognizes active Notch1 intracellular domain. The order of the samples were the same as that in Figure 3b except that lane 1 is the new negative control sample.

Recent studies showed that the proteasome inhibitory activity as well as the cell killing effects of bortezomib and MG132 could be specifically blocked by two antioxidants, tiron and edaravone, respectively [24,25]. As Z-LLNle-CHO is structurally similar to MG132, we speculated that edaravone might also be able to reverse the cytotoxicity of Z-LLNle-CHO by blocking its proteasome inhibition activity. Therefore, we first treated MCF-7 and MDA-MB-231 cells with different combinations of bortezomib or Z-LLNle-CHO and tiron or edaravone, and then measured cell growth using the MTS assay. Consistent with previous studies, tiron but not edaravone rescued cells from bortezomib-induced cell killing. Most importantly, we found edaravone but not tiron could rescue cells from Z-LLNle-CHO-induced cell killing (Figure 5a).

Next, we tested whether or not edaravone could rescue proteasome activity from Z-LLNle-CHO-induced inhibition. We exposed cells to edaravone at the concentration that showed best cell growth rescue in the presence of Z-LLNle-CHO and measured proteasome activity using the three approaches we used above. We used tiron to reverse bortezomib-induced proteasome inhibition as a control. We found that edaravone indeed rescued the proteasome activity from Z-LLNle-CHO-induced, but not bortezomib-induced, inhibition. Although the proteasome activity of edaravone rescued from Z-LLNle-CHO-induced inhibition was not to the same extent as tiron rescued bortezomib-induced inhibition in the cell based proteasome assay (Figure 5b), the rescued proteasome activity was enough to prevent the accumulation of polyubiquitinated proteins (lane 4 compared with lane 2 in Figure 3b) and redistribution of cellular ubiquitin (Figure 3c, treatment 4 *vs*. treatment 2). In addition, we found edaravone also partially restored γ-secretase activity from Z-LLNle-CHO-induced inhibition (Figure 5c).

### γ-secretase inhibition activity of Z-LLNle-CHO does not contribute to its cytotoxicity to breast cancer cells

**Figure 6:**
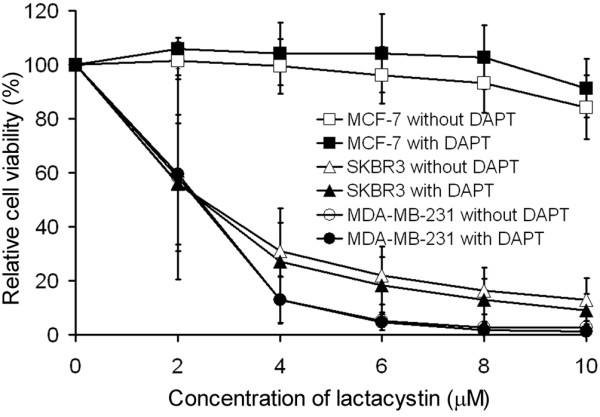
No additive effect from the combination of γ-secretase inhibition and proteasome inhibiton. Cells were exposed to increasing concentrations of lactacystin with or without 5 μM of DAPT for 72 hours and cell growth was measured by MTS assay. Results represent the mean ± standard deviation of three independent experiments.

To investigate whether the cytotoxicity of Z-LLNle-CHO to breast cancer cells is due to the summation or synergy of its dual activities, we tested whether a combination of a specific γ-secretase inhibitor with a specific proteasome inhibitor could produce an additive or synergetic effect on cell killing. We subjected cells to increasing concentrations of lactacystin with or without 5 μM of DAPT that completely inhibited γ-secretase activity in the cell lines tested. We found the dose-response curves of individual cell lines treated with or without DAPT was almost identical (Figure 6), which suggests there was no additive or synergetic effects of inhibiting both γ-secretase activity and proteasome activity. Therefore, γ-secretase inhibitory activity of Z-LLNle-CHO most likely does not contribute to its cell killing effect in breast cancer cells.

## Discussion

Blocking Notch signaling by inhibiting γ-secretase activity with small molecules has been suggested to be a promising approach to battle breast cancer [13,14]. In fact, there are three ongoing clinical trials registered at ClinicalTrials.gov using GSIs in the treatment of breast cancer. However, experimental data supporting the effectiveness of GSIs in the inhibition of cell growth or killing of breast cancer cells have been scarce. Two recent reports, however, have now shown that Z-LLNle-CHO, commonly called GSI I, has such an effect both *in vitro *and *in vivo *[15,16].

In the present study, we first compared the cytotoxicity and activity of Z-LLNle-CHO with two other popularly used GSIs, DAPT and L-685,458. We found that completely inhibiting γ-secretase activity by DAPT and L-685,458 had no effect on cell viability and proliferation of a panel of six breast cancer cell lines with different genetic backgrounds. In contrast, Z-LLNle-CHO could cause cell death even at concentrations that did not completely inhibit γ-secretase activity. Therefore, we conclude that the cell killing effect of Z-LLNle-CHO on breast cancer cells is not mediated by γ-secretase inhibition.

We next measured the proteasome inhibition potential of Z-LLNle-CHO. In contrast to two previous reports that Z-LLNle-CHO at concentrations that inhibited cell growth did not significantly inhibit proteasome activity (see supplemental materials in [15,26]), we found that it could inhibit proteasome activity by about 50% in intact cells even at a concentration that did not show significant cytotoxicity in two cell lines tested. Our result is consistent with a recent study that was published during the revision of this manuscript [27]. The new study showed that Z-LLNle-CHO at about 0.3 μM (calculated by us based on scale) inhibited proteasome activity by 80% and slowed cell growth by 20% in MCF-7 cells. As the approach the new study used to measure proteasome activity is different from ours, the extent of proteasome activity inhibition cannot be compared between their data and ours. However, both studies show that Z-LLNle-CHO could significantly inhibit proteasome activity at concentrations that showed dose-dependent cytotoxicity. The previous two studies used the same method to measure proteasome activity as the latest study but differed from ours. Therefore, it is easy to explain the discrepancy between their data and ours but we cannot explain the discrepancy between their data and the latest study.

Furthermore, we found that the relative cellular sensitivity of six breast cancer cell lines to Z-LLNle-CHO was the same as that to three widely used but structurally unrelated proteasome inhibitors and was also consistent with a previous study [23]. This consistency strongly suggests that the cell killing effect of Z-LLNle-CHO is due to its proteasome inhibitory function. Most convincingly, we found that the cytotoxic effect of Z-LLNle-CHO could be reversed by a specific antioxidant that blocked its proteasome inhibitory activity. Finally, we tested but did not find any additive effect of the combination of a specific γ-secretase inhibitor and a specific proteasome inhibitor on breast cancer cell growth. Therefore, we conclude that the cytotoxicity of Z-LLNle-CHO to breast cancer cells is mediated by proteasome inhibition.

We noticed that edaravone treatment also partially rescued γ-secretase activity from Z-LLNle-CHO-induced inhibition. However, because inhibition of γ-secretase alone or in combination with proteasome inhibition had no effect on cell survival/proliferation or cellular response to proteasome inhibition, we do not consider partially restored γ-secretase activity as a major contributor to the reversion of the cytotoxicity induced by Z-LLNle-CHO. Likewise, although edaravone has been reported to protect cells from apoptosis by acting as an antioxidant [28,29], we do not think its free radical scavenging activity is a major contributor because it had no effect on bortezomib-induced cytotoxicity. Therefore, its ability to restore proteasome activity through unknown mechanism(s) most likely accounts for the reversion of the cytotoxicity of Z-LLNle-CHO.

Both previous studies used transient transfection of N1ICD to rescue the cell death induced by Z-LLNle-CHO treatment and argued that the reversion of the phenotype by N1ICD transfection indicated that Z-LLNle-CHO induced cell death through inhibiting Notch signaling pathway [15,16]. However, transient overexpression of N1ICD has been reported to inhibit wild-type p53-induced apoptosis in immortalized epidermal cells [30], to inhibit dexamethasone, etoposide, or Fas-ligand-induced apotosis in mature T-cells [31], and to protect H460 (lung cancer), HepG2 (liver cancer), and HT1080 (fibrosarcoma) from several chemotherapy drugs [32]. Therefore, an alternative interpretation of the data from the two previous studies is that N1ICD over-expression provided pro-survival signals that antagonize the pro-apoptotic effects of Z-LLNle-CHO.

It is worthy noting that many of the effects of Z-LLNle-CHO reported in previous studies, including G2/M arrest and regulation of apoptosis-related protein, are consistent with the reported effects of other proteasome inhibitors [33-37]. In addition, similar to the additive effects of 4-OH-TAM and Z-LLNle-CHO on the inhibition of T47D:A18 cells growth [15], additive or even synergistic effects have also been reported between tamoxifen and bortezomib in some but not all ER-positive breast cancer cell lines tested [38,39]. Although the similarities between the biological effects of Z-LLNle-CHO and those of other proteasome inhibitors do not necessarily mean that they function the same, our finding that Z-LLNle-CHO inhibits breast cancer cell growth as a proteasome inhibitor can explain the data produced with the use of Z-LLNle-CHO in previous studies.

It should be pointed out that although the latest study by Rasul and colleagues found that Z-LLNle-CHO has proteasome inhibitory function at concentrations that showed dose-dependent cytotoxicity [27], the authors did not consider its proteasome inhibitory activity as the major contributor to its cell killing effects because the cytotoxicity of Z-LLNle-CHO and MG132 was 'markedly different', although their proteasome inhibition potential was similar. However, by careful analysis of their data, we found that the proteasome inhibition potentials of Z-LLNle-CHO and MG132 differed by more than two-fold, not less than the difference in cytotoxicity, within the range of concentrations that Z-LLNle-CHO and MG132 showed 'markedly different' cytotoxicity (below 0.6 μM). Most importantly, Z-LLNle-CHO at 0.75 μM in their study slowed MCF-7 cell growth by 80%, but only inhibited γ-secretase activity by 25%. Meanwhile, it inhibited proteasome activity by 80%. Therefore, their data is more consistent with our conclusion that the cytotoxicity of Z-LLNle-CHO was not due to γ-secretase inhibition, but due to proteasome inhibition.

The observation that both Z-LLNle-CHO and MG132 at given concentrations inhibited proteasome activities to comparable levels in MCF-7 and MDA-MB-231 cells, but showed different cytotoxicity, is not surprising because this has also been observed for bortezomib [23]. The reduced sensitivity of ER-positive MCF-7 cells may be a consequence of pro-survival signal provided by the ER signaling pathway in these ER-positive breast cancer cells. This hypothesis is consistent with the observed additive or even synergistic effect between tamoxifen and Z-LLNle-CHO or bortezomib. However, this requires further investigation. Regardless of the mechanisms, our results, together with the previous reports, suggest that the future clinical trials testing the effectiveness of proteasome inhibitors in treating breast cancer should take the ER status into consideration when enrolling patients.

The observation that two specific GSIs, DAPT and L-685,458, had no effect on the survival and proliferation of breast cancer cells does not eliminate the potential use of GSIs or other approaches to block Notch signaling for breast cancer treatment. The results presented here were obtained from *in vitro *cell culture experiments. The effects of GSIs on the tumors grown *in vivo*, where the Notch signaling might be more active due to enhanced ligand-receptor interaction, could be different and need further investigation. Alternatively, these drugs might block the signaling pathway of some as yet unidentified substrate(s) which antagonizes the effect of reduced Notch1 signaling on breast cancer cell survival and proliferation. There are at least a dozen known γ-secretase substrates and most of the available GSIs have no preference for specific substrates. Rather than laboriously testing all potential candidates that antagonize Notch1, it might be better to develop substrate-specific GSIs. To this end, it is encouraging to note that compounds that can preferentially modulate γ-secretase activity against Aβ42 over Notch have recently been reported [40]. These compounds target the substrate (Aβ42) rather than the γ-secretase active site itself. In principle, it should also be possible to find drugs that target individual Notch homologs. Alternatively, it might be useful to develop neutralizing antibody against individual Notch homologs just as the trastuzumab targets HER2/neu.

Furthermore, the results of this study do not diminish the potential use of Z-LLNle-CHO for breast cancer treatment. In fact, we believe that clarifying its role as a proteasome and γ-secretase dual inhibitor will help to direct its potential development for clinical use. However, we do caution that results obtained using Z-LLNle-CHO as the sole GSI to study the biological outcomes of blocking Notch signaling [41-43] should be interpreted cautiously or reproduced using more specific GSIs.

## Conclusions

The present study demonstrated that the cytotoxicity of Z-LLNle-CHO toward breast cancer cells was not mediated by γ-secretase inhibiton as reported previously, but by proteasome inhibition. This clarification might help its potential development as a chemotherapeutic agent. The results presented also call for careful interpretation of data produced with using Z-LLNle-CHO as the sole γ-secretase inhibitor.

## Abbreviations

DMEM: Dulbecco's modified eagle's medium; DMSO: dimethyl sulfoxide; ER: estrogen receptor; FBS: fetal bovine serum; GSI: γ-secretase inhibitor; N1ICD: Notch1 intracellular domain; N1EXT: Notch1 extracellular truncation; PBS: phosphate-buffered saline; PCR: polymerase chain reaction; SD: standard deviation.

## Competing interests

The authors declare that they have no competing interests.

## Authors' contributions

JH participated in the conception of the study and its design, performed most of the experiments, and wrote the first draft of the manuscript. IM performed cell viability/proliferation assays. MJH and JAT participated in the conception of the study and its design and drafted the final manuscript. All authors read and approved the final manuscript.
